# Unveiling difficult-to-treat rheumatoid arthritis: long-term impact of biologic or targeted synthetic DMARDs from the KOBIO registry

**DOI:** 10.1186/s13075-023-03165-w

**Published:** 2023-09-19

**Authors:** Ju-Yang Jung, Eunyoung Lee, Ji-Won Kim, Chang-Hee Suh, Kichul Shin, Jinhyun Kim, Hyoun-Ah Kim

**Affiliations:** 1 Department of Rheumatology, Ajou University School of Medicine, 164 World Cup-Ro, Yeongtong-Gu, Suwon, 16499 Korea; 2 Department of Neurology, McGovern Medical School, University of Texas Health Science Center at Houston, Texas, USA; 3 Department of Internal Medicine, College of Medicine, Seoul National University, Seoul, Korea; 4 Division of Rheumatology, Department of Internal Medicine, College of Medicine, Chungnam National University, Daejeon, Korea

**Keywords:** Rheumatoid arthritis, Difficult-to-treat, Biologic therapy, DMARDs

## Abstract

**Background:**

While the availability of biological or targeted synthetic disease-modifying anti-rheumatic drugs (b/tsDMARDs) has improved outcomes for rheumatoid arthritis (RA) patients, there remains a subset of individuals who fail to achieve low disease activity or remission despite multiple cycles of b/tsDMARDs. This state is referred to as 'difficult-to-treat (D2T)' RA.

**Methods:**

Data from the Korean College of Rheumatology Biologics registry were utilized to analyze patients with RA who were treated with b/tsDMARDs.

**Results:**

Among 2,321 RA patients with RA treated with b/tsDMARDs, 271 (11.7%) were diagnosed with D2T RA. Lower age (OR = 0.98, *p* < 0.001), longer disease duration (OR = 1.06, *p* < 0.001), lower patient global assessment (OR = 0.89, *p* = 0.045), higher SDAI (OR = 1.06, *p* = 0.014) and RAPID3 (OR = 1.06, *p* = 0.002), lower RF positivity (OR = 0.65, *p* = 0.04), and lower prior use of methotrexate (OR = 0.44, *p* = 0.008), sulfasalazine (OR = 0.59, *p* = 0.003), and leflunomide (OR = 0.67, *p* = 0.013) were associated with D2T RA. The drug survival rate of b/tsDMARDs did not differ between patients with D2T RA and non-D2T RA (*p* = 0.35). However, the drug survival of individual b/tsDMARD differed between patients with D2T RA and non-D2T RA after eight years. Patients with D2T RA withdrew from b/tsDMARDs due to inefficacy more frequently than those without D2T RA (*p* < 0.001).

**Conclusions:**

D2T RA patients experienced higher disease activity despite maintaining b/tsDMARD therapy. Withdrawal rates due to inefficacy were higher in D2T RA. Effective therapeutic strategies are needed to improve disease control and treatment outcomes in this unique patient population.

**Supplementary Information:**

The online version contains supplementary material available at 10.1186/s13075-023-03165-w.

## Introduction

The availability of biologic disease-modifying anti-rheumatic drugs (bDMARDs) and targeted synthetic disease-modifying anti-rheumatic drugs (tsDMARDs) has established treat-to-target strategies in rheumatoid arthritis (RA), leading to improved outcomes. These treatments have enabled more patients to achieve and maintain remission or low disease activity, providing protection against joint deformities and severe complications compared to previous decades. However, despite multiple cycles of biologics or tsDMARDs, there are still RA patients who fail to reach the treatment target, and this condition has recently been termed "difficult-to-treat (D2T) RA" [1, 2]. The European League Against Rheumatism (EULAR) has provided a definition for D2T RA, which refers to cases where a patient has active or progressive disease while receiving multiple biologics or tsDMARDs with different mechanisms of action [3]. The prevalence of D2T RA varies depending on the characteristics of the study population and has been reported to range from 5 to 20% of all RA patients.

The concept of “difficult” in D2T RA indicates a state of refractory disease where traditional DMARDs are ineffective. However, it is important to note that the term also encompasses other factors that contribute to the difficulty in achieving treatment success. These factors include limited DMARD options due to adverse events or comorbidities, as well as non-adherence to prescribed treatments [4]. In an international survey conducted to establish the definition of D2T RA, several prominent characteristics were identified. These included the presence of interfering comorbidities, extra-articular manifestations of RA, radiographic progression indicating joint damage, the presence of synovitis as defined by ultrasonography, and significant side effects from previous treatments [5, 6]. Recently, the EULAR addressed the considerations and research agendas pertaining to D2T RA [7]. The EULAR raised several important questions related to D2T RA, such as identifying suspected contributing factors including smoking and obesity, understanding the mechanisms underlying the failure of biologic and tsDMARDs to suppress inflammation in D2T RA, and evaluating the utility of methods like synovial biopsies in selecting appropriate treatment options. Additionally, the assessment of comorbidities and patient compliance with prescribed medications was emphasized. Despite these inquiries, there is still a lack of comprehensive data regarding the proportion of “true” refractory D2T RA and the efficacy and safety of b/tsDMARDs in this specific patient population. To date, there is a scarcity of references that comprehensively outline the clinical characteristics of D2T RA and propose effective management strategies. It is crucial to address this knowledge gap in order to improve treatment outcomes and enhance the overall management of patients facing the challenges of D2T RA.

Therefore, this study aimed to examine the overall treatment outcomes, including drug survival, in patients with D2T RA who received b/tsDMARDs. We attempted to identify the clinical characteristics associated with D2T RA by analysing data from a nationwide and prospective cohort. The study evaluated the effectiveness, safety, and rates of drug discontinuation or switching among patients with D2T and non-D2T RA who were treated with b/tsDMARDs.

## Materials and methods

### Data collection

We obtained the data from the Korean College of Rheumatology Biologics (KOBIO) Registry, an ongoing, nationwide, multi-center, prospective cohort [8], comprising patients with RA, ankylosing spondylitis, and psoriatic arthritis. All enrolled patients were aged 18 years or older and had initiated or switched to a new biologic agent or tsDMARDs for their respective conditions. Assessments were conducted by rheumatologists after obtaining consent from each patient. Informed consent forms and study protocols were approved by the independent institutional review boards and ethics committees of each participating centre. Ethics approval for the KOBIO registry was provided by the institutional review boards (IRBs) of all the 58 participating institutions. The current study also received ethics approval from the IRB of the researchers' affiliated hospitals (AJOUIRB-DB-2022–362). This study was conducted in accordance with the principles outlined in the Declaration of Helsinki, and all the patients provided written informed consent to participate.

The diagnosis of RA in the enrolled patients was made based on either the 1987 American College of Rheumatology (ACR) or the 2010 ACR/EULAR criteria [9, 10]. We collected various data on patients with RA, including demographic information, comorbidities, presence of extra-articular manifestations, laboratory results such as erythrocyte sedimentation rate (ESR), C-reactive protein (CRP), rheumatoid factor (RF) and anti-cyclic citrullinated peptide (CCP) antibody positivity, and disease activity indicators such as disease activity score (DAS) 28 and Simplified Disease Activity Index (SDAI). Medication data encompassed the use of conventional synthetic (cs) DMARDs, glucocorticoids (GCs), bDMARDs, and tsDMARDs. Additionally, we collected information on the presence of radiographic erosions, routine assessment of patient index data 3 (RAPID3), drug discontinuation and switching, and adverse events. Treatment outcomes were assessed at the 1-year follow-up and included measurements of DAS28 and ACR20/50/70 responses.

### Study design

All patients with RA were divided into two groups: D2T and non-D2T RA, according to the criteria defined by the EULAR when they started new b/tsDMARDs [2]. The EULAR definition of D2T RA requires patients to have received two or more different b/tsDMARDs with distinct mechanisms of action, after failing to respond to csDMARDs. Additionally, patients must meet at least one of the following criteria: moderate disease activity, defined as a DAS28-ESR > 3.2 or Clinical Disease Activity Index (CDAI) > 10; presence of signs and/or symptoms suggestive of active disease; inability to taper GC treatment below a dose of 7.5 mg/day prednisone or equivalent; rapid radiographic progression; and RA symptoms that significantly impair the patient's quality of life. Due to the limitations of the data obtained from the KOBIO Registry, the classification into D2T and non-D2T RA groups was based on confirming moderate disease activity as a DAS28-ESR > 3.2 or Clinical Disease Activity Index (CDAI) > 10 and the inability to taper GC treatment below a dose of 7.5 mg/day prednisone or equivalent, as defined by the EULAR criteria. Various clinical data, including laboratory findings, disease activity markers, and medication history, were compared and analyzed between the D2T and non-D2T RA groups.

### Comparison of treatment outcomes

All the patients were categorized into the groups based on each drug started after being defined as D2T and non-D2T RA, regardless of previously used drugs. Based on the data collected throughout the follow-up period, the rates of drug retention, withdrawal, and switching were compared between the D2T and non-D2T groups. The efficacy of biologics or tsDMARDs in the D2T and non-D2T groups was assessed by evaluating DAS28, SDAI, CDAI, and ACR20/50/70 responses during the follow-up period.

### Statistical analysis

Data were expressed as mean ± standard deviation or median with interquartile range as appropriate, or frequency (percentage). To compare D2T and non-D2T RA patients, Pearson’s χ2 test or t-test was used. The χ2 test and independent t-test were employed for analyzing categorical and continuous variables, respectively. The normality of the data was assessed using the Shapiro–Wilk test. Univariate and multivariate regression analyses were conducted with clinical variables to evaluate their association with D2T RA. The factors for multivariate analysis were chosen based on previous data (age, BMI, smoker, and use of corticosteroid) as well as the outcomes of univariate analysis, which encompassed various factors such as disease duration and comorbidities. The drug retention rate was determined using Kaplan–Meier analysis, and group comparisons were made using the log-rank test. In the Kaplan–Meier curves of b/tsDMARDs, the patients were categorized into the groups based on each drug started after being defined as D2T RA, regardless of previously used drugs. The curve depicted the period from the initiation of the specific drug until its discontinuation or switching. Statistical analysis was performed using SPSS version 25.0 software (IBM Corporation, Armonk, NY, USA). A *p*-value less than 0.05 (two-tailed) was considered statistically significant.

## Results

### Comparison of clinical characteristics between D2T and non D2T-RA patients

The mean age of the total RA patient population was 54.6 years (± 12.8). In the D2T RA group, the mean age was 53.8 years (± 12.4), while in the non-D2T group, it was 54.8 years (± 12.9) (*p* = 0.24) (Table 1). The percentage of male patients was 17.5% in the overall population, 14.4% in the D2T group, and 18% in the non-D2T group. There were no significant differences in body mass index (BMI) or the proportion of smoking patients between the two groups. Regarding comorbidities, 12.5% of patients had diabetes, 30.3% had hypertension, and 0.4% had cancer, with no significant differences between the D2T and non-D2T groups. However, the percentage of patients with cardiovascular disease (CVD) was 25.7%, which was higher in the D2T group (32.5%) compared to the non-D2T group (24.8%, *p* = 0.007). In terms of extra-articular manifestations, 6% of patients had interstitial lung disease, 3.1% had secondary Sjögren's syndrome, and 2.4% had subcutaneous rheumatoid nodules. The proportion of patients with subcutaneous rheumatoid nodules was higher in the D2T group (4.4%) compared to the non-D2T group (2.1%, *p* = 0.018).

**Table 1:** Comparisons of demographic and clinical characteristics between D2T and non-D2T groups in KOBIO registry

**Variable**	**Total (*n* = 2,321)**	**D2T (*n* = 271)**	**Non-D2T (*n* = 2,050)**	*p-*value
**Demographics**				
Age, years, mean (SD)	54.6 (12.8)	53.8 (12.4)	54.8 (12.9)	0.240
Sex, N (%)				0.148
Male	407 (17.5)	39 (14.4)	368 (18.0)	
Female	1,914 (82.5)	232 (85.6)	1,682 (82.1)	
BMI, mean (SD)	22.8 (3.5)	22.7 (3.7)	22.8 (3.5)	0.632
Smoking, N (%)				0.973
Ex-smoker	215 (9.3)	26 (9.6)	189 (9.2)	
Current smoker	185 (8.0)	21 (7.8)	164 (8.0)	
Never	1,921 (82.8)	224 (82.7)	1,697 (82.8)	
**Comorbidities**				
Diabetes mellitus, N (%)	290 (12.5)	33 (12.2)	257 (12.5)	0.866
Hypertension, N (%)	703 (30.3)	83 (30.6)	620 (30.2)	0.897
Cardiovascular disease, N (%)	597 (25.7)	88 (32.5)	509 (24.8)	**0.007**
Cancer, N (%)	10 (0.4)	2 (0.7)	8 (0.4)	0.329
**Extraarticular manifestations**				
Scleritis or episcleritis, N (%)	5 (0.2)	0 (0.0)	5 (0.2)	-
Secondary Sjogren’s syndrome, N (%)	71 (3.1)	5 (1.9)	66 (3.2)	0.217
Subcutaneous rheumatoid nodule, N (%)	55 (2.4)	12 (4.4)	43 (2.1)	**0.018**
Cutaneous vasculitis, N (%)	4 (0.2)	1 (0.4)	4 (0.2)	0.393
Pleuritis, N (%)	9 (0.4)	1 (0.4)	8 (0.4)	-
Interstitial lung disease, N (%)	140 (6.0)	22 (8.1)	118 (5.8)	0.128
**Disease status**				
Disease duration, years, median [IQR]	5.6 [1.8, 11.9]	9.7 [5.2, 15.2]	5 [1.6, 11.2]	**< 0.001**
RF positivity, N (%)	1,854 (82.9)	201 (79.1)	1,653 (83.4)	0.089
Anti-CCP Ab positivity, N (%)	1,671 (85.8)	170 (84.6)	1,501 (85.9)	0.606
Tender joint count, median [IQR]	7 [4, 12]	8 [4, 14]	7 [4, 11]	0.073
Swollen joint count, median [IQR]	6 [3, 9]	6 [3, 10]	5 [2, 9]	0.144
Patient global assessment, mean (SD)	6.83 (2.04)	7.01 (1.99)	6.81(2.04)	0.130
Physician global assessment, mean (SD)	6.47 (1.82)	6.71 (1.69)	6.44 (1.84)	**0.024**
ESR, mm/hr, median [IQR]	44 [28, 65]	47 [29, 75]	44 [28, 65]	0.055
CRP, mg/dL, median [IQR]	1.25 [0.43, 2.93]	1.72 [0.34, 3.78]	1.21 [0.44, 2.80]	**0.033**
DAS28-ESR, mean (SD)	5.52 (1.14)	5.65 (1.15)	5.51 (1.13)	0.062
DAS28-CRP, mean (SD)	4.83 (1.13)	4.98 (1.16)	4.81 (1.12)	**0.020**
SDAI, mean (SD)	28.9 (11.8)	31.1 (12.9)	28.6 (11.6)	**0.003**
CDAI, mean (SD)	26.7 (11.0)	28.2 (11.9)	26.4 (10.9)	**0.02**
Radiographic erosions, N (%)	889 (38.3)	100 (36.9)	789 (38.5)	0.613
**Function**				
RAPID3, mean (SD)	15.3 (5.7)	16.8 (5.5)	15.1 (5.6)	**< 0.001**

The data are included at starting new b/tsDMARDs. Data were expressed as mean ± standard deviation (SD) or median with interquartile range (IQR) as appropriate, or frequency (percentage). *P*-values are calculated using chi square test, Student’s t-test, or Wilcoxon rank-sum test. Bold values indicate significant *p*-values

*RA* rheumatoid arthritis, *KOBIO* Korean Rheumatology Biologics registry, *BMI* body mass index, *RF* rheumatoid factor, *Anti-CCP Ab* anti-citrullinated protein antibody, *ESR* erythrocyte sedimentation rate, *CRP* C-reactive protein, *DAS* disease activity score, *VAS* visual analogue scale, *ILD* interstitial lung disease, *csDMARDs* conventional synthetic disease modifying anti-rheumatic drugs, *IQR* inter-quartile range, *TNF* tumor necrosis factor, *D2T* difficult to treat

The disease status at the time of enrollment in the KOBIO registry was compared between groups. The median disease duration was longer in the D2T group (9.73 [5.18 – 15.24] years) compared to the non-D2T group (5.03 [1.57–11.15] years, *p* < 0.001). The proportions of RF and anti-CCP antibody positivity did not differ significantly between the D2T and non-D2T groups (79.1 vs. 83.4%, *p* = 0.089 and 84.6 vs. 85.9%, *p* = 0.606). While the tender and swollen joint counts and patient global assessment were similar between the D2T and non-D2T groups, the physician global assessment was higher in the D2T group (6.71 ± 1.69) compared to in the non-D2T group (6.44 ± 1.84, *p* = 0.024). ESR level was slightly higher in the D2T group (47 [29–75]) compared to the non-D2T group (44 [28—65], *p* = 0.055), and CRP was higher in the D2T group (1.72 [0.34 – 3.78]) than in the non-D2T group (1.21 [0.44 – 2.8], *p* = 0.033). The DAS28-CRP was higher in the D2T group (4.98 ± 1.16) than in the non-D2T group (4.81 ± 1.12, *p* = 0.02). The SDAI and CDAI were also higher in the D2T group (31.1 ± 12.9 and 28.2 ± 11.9, respectively) compared to the non-D2T group (28.6 ± 11.6 and 26.4 ± 10.9, respectively, *p* = 0.003 and 0.02). The RAPID3 was higher in the D2T group (16.8 ± 5.5) compared to the non-D2T group (15.1 ± 5.6, *p* < 0.001).

### Comparisons of management between D2T and non-D2T RA patients

In previous treatments, the proportion of patients on methotrexate, sulfasalazine, and leflunomide were lower in the D2T group (85.6%, 29.2%, and 43.5%, respectively) compared to the non-D2T group (94.8%, 39.5%, and 53.4%, respectively; *p* < 0.001, 0.001, and 0.002, respectively) (Table 2). As concurrent treatments, the proportion of patients using methotrexate, sulfasalazine, and leflunomide were lower in the D2T group (73.8%, 7.8%, 12.2%) than in the non-D2T group (82.2%, 14.0%, 27.5%, respectively; *p* < 0.001, 0.004, < 0.001). The proportions of patients using corticosteroids and their dosages did not differ between the D2T and non-D2T groups.

**Table 2:** Comparisons of medication and withdrawal of biologics between D2T and non-D2T groups in KOBIO registry

**Variable**	**Total (*n* = 2,321)**	**D2T (*n* = 271)**	**Non-D2T (*n* = 2,050)**	*p-*value
**Medication**				
Previous treatments, N (%)				
Prior use of methotrexate	2,175 (93.7)	232 (85.6)	1,943 (94.8)	**< 0.001**
Prior use of sulfasalazine	889 (38.3)	79 (29.2)	810 (39.5)	**0.001**
Prior use of leflunomide	1,212 (52.2)	118 (43.5)	1,094 (53.4)	**0.002**
Concomitant treatments, N (%)				
Methotrexate	1,885 (81.2)	200 (73.8)	1,685 (82.2)	**0.001**
Sulfasalazine	308 (13.3)	21 (7.8)	287 (14.0)	**0.004**
Leflunomide	597 (25.7)	33 (12.2)	564 (27.5)	**< 0.001**
Corticosteroid use	1,975 (85.1)	229 (84.5)	1,746 (85.2)	0.757
Corticosteroid dosage^a^, mg/day, mean (SD)	5.4 (3.1)	5.7 (3.3)	5.4 (3.1)	0.101
Prior use of biologic agents, N (%)				**< .0001**
0	1,686 (72.6)	0 (0.0)	1,686 (82.2)	
1	362 (15.6)	0 (0.0)	362(17.7)	
≥ 2	273 (11.8)	271 (100.0)	2 (0.1)	
Number of prior biologic agents, median [IQR]	0 [0,1]	2 [2, 3]	0 [0,0]	**< 0.001**
Current bDMARDs or tsDMARDs type, N (%)				**< 0.001**
TNF inhibitors	1,064 (45.9)	53 (19.6)	1,011 (49.3)	
Etanercept	314 (29.5)	10 (18.9)	304 (30.1)	
Infliximab (remsima + remicade)	210 (19.7)	10 (18.9)	200 (19.8)	
Adalimumab	379 (35.6)	16 (30.2)	363 (35.9)	
Golimumab	161 (15.1)	17 (32.1)	144 (14.2)	
Rituximab	27 (1.2)	19 (7.0)	8 (0.4)	
Abatacept	305 (13.2)	37 (13.7)	268 (13.1)	
Tocilizumab	562 (24.2)	91 (33.7)	471 (23)	
JAK inhibitors	362 (15.6)	70 (25.9)	292 (14.2)	
Tofacitinib	206 (56.9)	50 (71.4)	156 (53.4)	
Baricitinib	136 (37.6)	17 (24.3)	119 (40.8)	
Upadacitinib	20 (5.5)	3 (4.3)	17 (5.8)	
**Withdrawal**				
Treatment duration, years, median [IQR]	3.74 [1.98, 5.48]	3.67 [1.91, 5.21]	3.76 [1.99, 5.53]	0.257
Withdrawal, N (%)	1,025 (44.2)	122 (45)	903 (44.1)	0.763
Discontinuation, N (%)	594 (25.6)	63 (23.3)	531 (25.9)	0.347
Switching, N (%)	591 (25.5)	85 (31.4)	506 (24.7)	**0.018**
Withdrawal reason				
Clinical remission, N (%)	74 (7.2)	14 (11.5)	60 (6.6)	0.053
Inefficacy, N (%)	445 (43.4)	71 (58.2)	374 (41.4)	**< 0.001**
Adverse events, N (%)	340 (33.2)	44 (36.1)	296 (32.8)	0.469
Other reasons, N (%)	293 (28.6)	19 (15.6)	274 (30.3)	**0.001**
**Death**				
Death, N (%)	53 (2.3)	10 (3.7)	43 (2.1)	0.099
Mortality duration, years, median [IQR]	1.2 [0.36, 3.47]	2.01 [0.25, 3.70]	1.1 [0.36, 3.41]	0.989

Data were expressed as mean ± standard deviation (SD) or median with interquartile range (IQR) as appropriate, or frequency (percentage). *P*-values are calculated using chi square test, Student’s t-test, or Wilcoxon rank-sum test. Bold values indicate significant *p*-values

*D2T* difficult to treat, *KOBIO* Korean College of Rheumatology Biologics & Targeted therapy, *csDMARDs* conventional synthetic disease modifying anti-rheumatic drugs, *IQR* inter-quartile range, *bDMARDs* biologic disease modifying anti-rheumatic drugs, *tsDMARDs* target synthetic disease modifying anti-rheumatic drugs, *TNF* tumor necrosis factor, *JAK* janus kinase

Biosimilars were included in each originators. ^a^prednisone-equivalent

### Risk factors for D2T RA with b/tsDMARDs

In univariable regression analysis, female sex (OR = 1.6, *p* = 0.033), disease duration (OR = 1.06, *p* < 0.001), physician global assessment (OR = 1.09, *p* = 0.037), DAS28-CRP (OR = 1.16, *p* = 0.031), SDAI (OR = 1.02, *p* = 0.002), CDAI (OR = 1.02, *p* = 0.023), RAPID3 (OR = 1.05, *p* < 0.001), prior use of methotrexate (OR = 0.36, *p* < 0.001) and sulfasalazine (OR = 0.36, *p* = 0.008), and concurrent use of methotrexate (OR = 0.57, *p* = 0.001) were found to be associated with D2T RA (Table 3). Multivariable regression analysis revealed that age (OR = 0.98, *p* < 0.001), disease duration (OR = 1.06, *p* < 0.001), patient global assessment (OR = 0.89, *p* = 0.045), SDAI (OR = 1.06, *p* = 0.014), RAPID3 (OR = 1.06, *p* = 0.002), and prior use of methotrexate (OR = 0.44, *p* = 0.008), sulfasalazine (OR = 0.59, *p* = 0.003), and leflunomide (OR = 0.67, *p* = 0.013) were associated with D2T RA.

**Table 3:** Logistic regression analysis for D2T RA among RA patients

Variable	Univariable		Multivariable	
	**OR (95% CI)**	***p****-value*	**Adjusted OR (95% CI)**	***p****-value*
Age	0.99 (0.98, 1.00)	0.180	0.98 (0.96, 0.99)	**0.001**
Sex, female	1.6 (1.03, 2.47)	**0.033**	1.72 (0.92, 3.20)	0.087
BMI	0.97 (0.93, 1.02)	0.247	1 (0.95, 1.04)	0.841
Current, ex- smoker	0.91 (0.61, 1.35)	0.608	1.37 (0.77, 2.43)	0.283
Disease duration	1.06 (1.04, 1.07)	**< 0.001**	1.06 (1.04, 1.08)	**< 0.001**
Patient global assessment	1.05 (0.97, 1.13)	0.21	0.89 (0.79, 1.00)	**0.045**
Physician global assessment	1.09 (1.01, 1.19)	**0.037**	1.1 (0.98, 1.23)	0.121
DAS28-ESR	1.14 (0.99, 1.30)	**0.055**	1.01 (0.72, 1.41)	0.973
DAS28-CRP	1.16 (1.01, 1.33)	**0.031**	0.79 (0.5, 1.26)	0.325
SDAI	1.02 (1.01, 1.03)	**0.002**	1.06 (1.01, 1.11)	**0.014**
CDAI	1.02 (1.00, 1.03)	**0.023**	0.97 (0.93, 1.01)	0.134
RAPID3	1.05 (1.03, 1.08)	**< 0.001**	1.06 (1.02, 1.1)	**0.002**
Comorbidities^a^	1.28 (0.96, 1.72)	0.084	1.32 (0.93, 1.87)	0.126
RF positivity	0.72 (0.50, 1.03)	0.073	0.65 (0.43, 0.98)	**0.040**
Anti-CCP Ab positivity	0.92 (0.61, 1.38)	0.698	1.17 (0.73, 1.88)	0.510
Prior use of methotrexate	0.36 (0.23, 0.57)	**< 0.001**	0.44 (0.24, 0.81)	**0.008**
Prior use of sulfasalazine	0.65 (0.47, 0.9)	**0.008**	0.59 (0.42, 0.83)	**0.003**
Prior use of leflunomide	0.76 (0.57, 1.02)	0.061	0.67 (0.49, 0.92)	**0.013**
Concomitant methotrexate	0.57 (0.40, 0.79)	**0.001**	0.74 (0.47, 1.16)	0.182
Concomitant corticosteroid	0.93 (0.62, 1.40)	0.610	0.87 (0.57, 1.34)	0.528

Values are calculated using logistic regression model. Bold values indicate significant *p*-values

*bDMARDs* biologic disease modifying anti-rheumatic drugs, *tsDMARDs* target synthetic disease modifying anti-rheumatic drugs, *D2T* difficult to treat, *OR* odds ratio, *CI* confidence interval, *BMI* body mass index, *DAS* disease activity score, *ESR* erythrocyte sedimentation rate, *CRP* C-reactive protein, *SDAI* simplified disease activity index, *CDAI* clinical disease activity index, *RAPID3* routine assessment of patient index data 3, *RF* rheumatoid factor, *Anti-CCP Ab* anti-citrullinated protein antibody, *csDMARDs* conventional-synthetic disease modifying anti-rheumatic drugs

^a^Comorbidities include hypertension, diabetes mellitus, cardiovascular diseases, and cancer

### Drug retention of b/tsDMARDs in D2T- and non-D2T RA patients

**Fig. 1:**
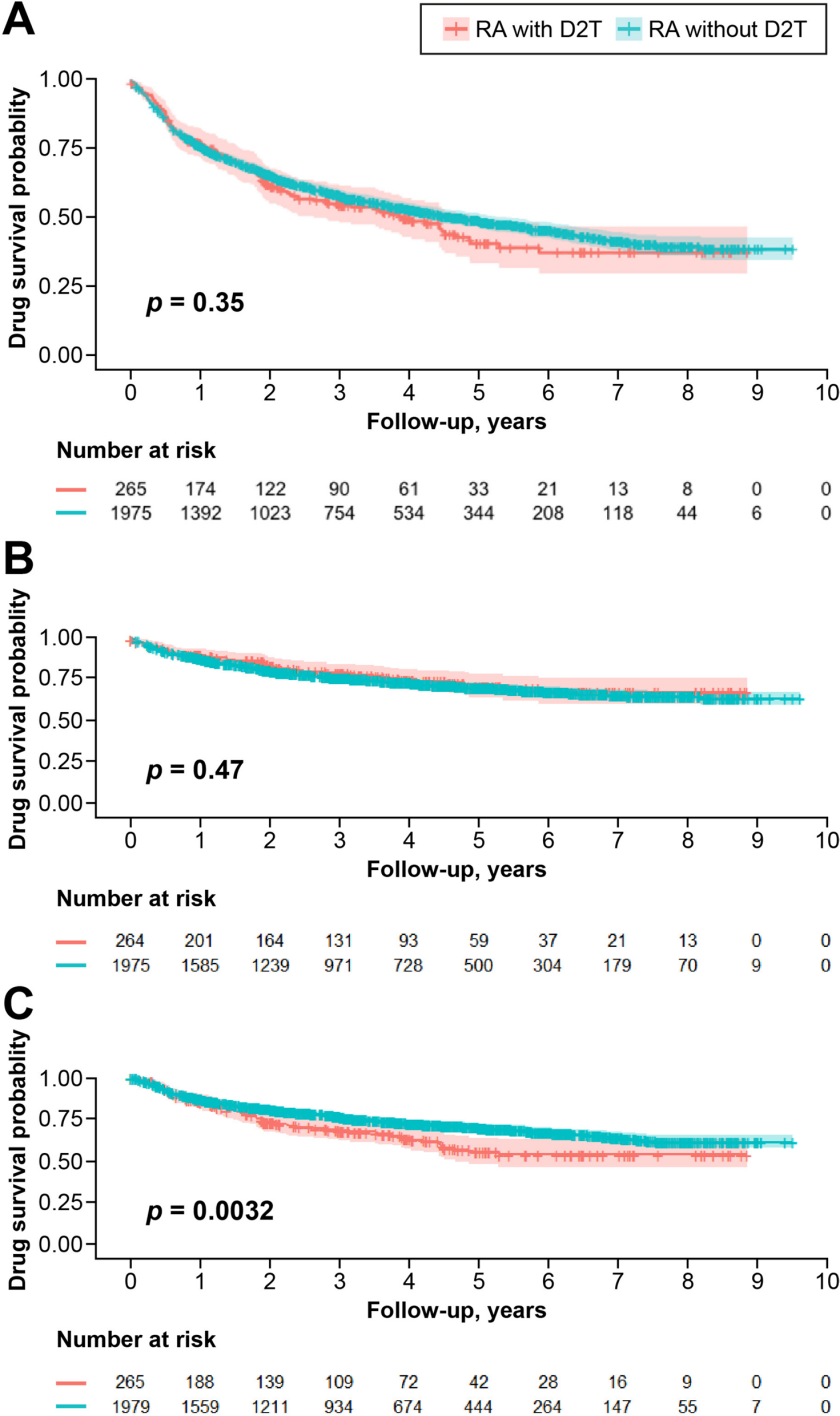
Comparison of b/tsDMARDs between D2T RA and non-D2T RA groups. **A** Comparison of drug retention between the D2T RA and non-D2T RA groups. **B** Comparison of drug discontinuation between the D2T RA and non-D2T RA groups. **C** Comparison of drug switching between the D2T RA and non-D2T RA groups

The drug retention rates did not show significant differences between the D2T and non-D2T groups (*p* = 0.35) (Fig. 1). There were no significant differences in drug discontinuation rates (*p* = 0.47), but there was a significant difference in drug switching rates between patients with D2T and non-D2T RA (*p* = 0.003). Mean observational period was 2.68 ± 2.22 years, 2.45 ± 2.15 years in D2T RA patients and 2.72 ± 2.23 years in non-D2T RA patients.

**Fig. 2:**
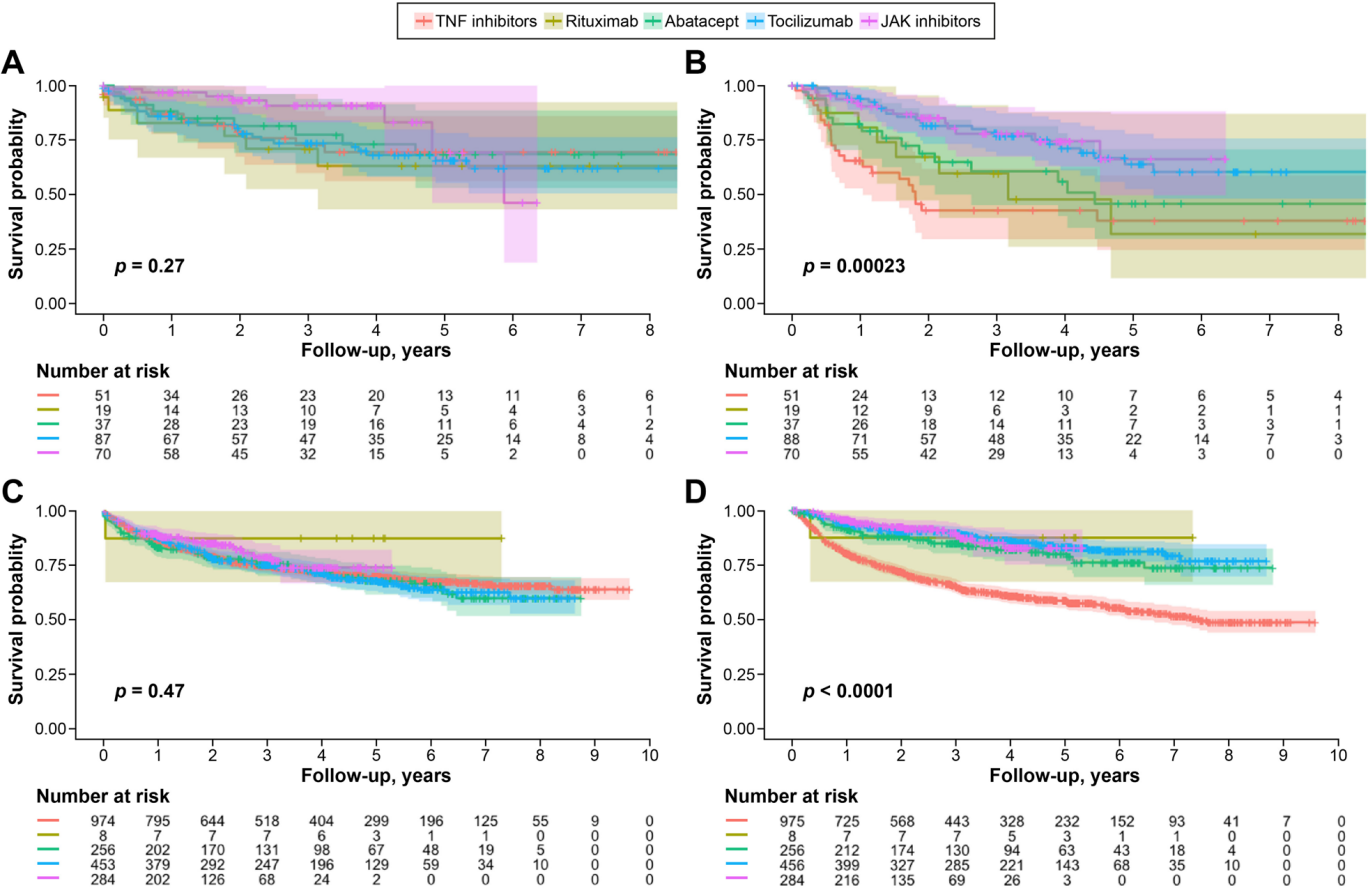
Kaplan–Meier curves for drug discontinuation and switching. **A** Comparison of drug discontinuation between b/tsDMARDs in patients with D2T RA. **B** Comparison of drug switching between b/tsDMARDs in patients with D2T RA. **C** Comparison of drug discontinuation between b/tsDMARDs in patients without D2T RA. **D** Comparison of drug switching between b/tsDMARDs in patients without D2T RA

The Kaplan–Meier curve analysis showed no significant differences in drug discontinuation rates for each b/tsDMARD (*p* = 0.27), while drug switching rates differed among Janus kinase (JAK) inhibitors, tocilizumab, abatacept, tumor necrosis factor (TNF) inhibitors, and rituximab (*p* < 0.001) in patients with D2T RA (Fig. 2). Additionally, in patients with non-D2T RA, there were no significant differences in drug discontinuation rates for each b/tsDMARD (*p* = 0.47), but drug switching rates differed in the order of rituximab, JAK inhibitors, tocilizumab, abatacept, and TNF inhibitors (*p* < 0.001).

### Comparison of efficacy of b/tsDMARDs between D2T and non-D2T RA patients

**Fig. 3:**
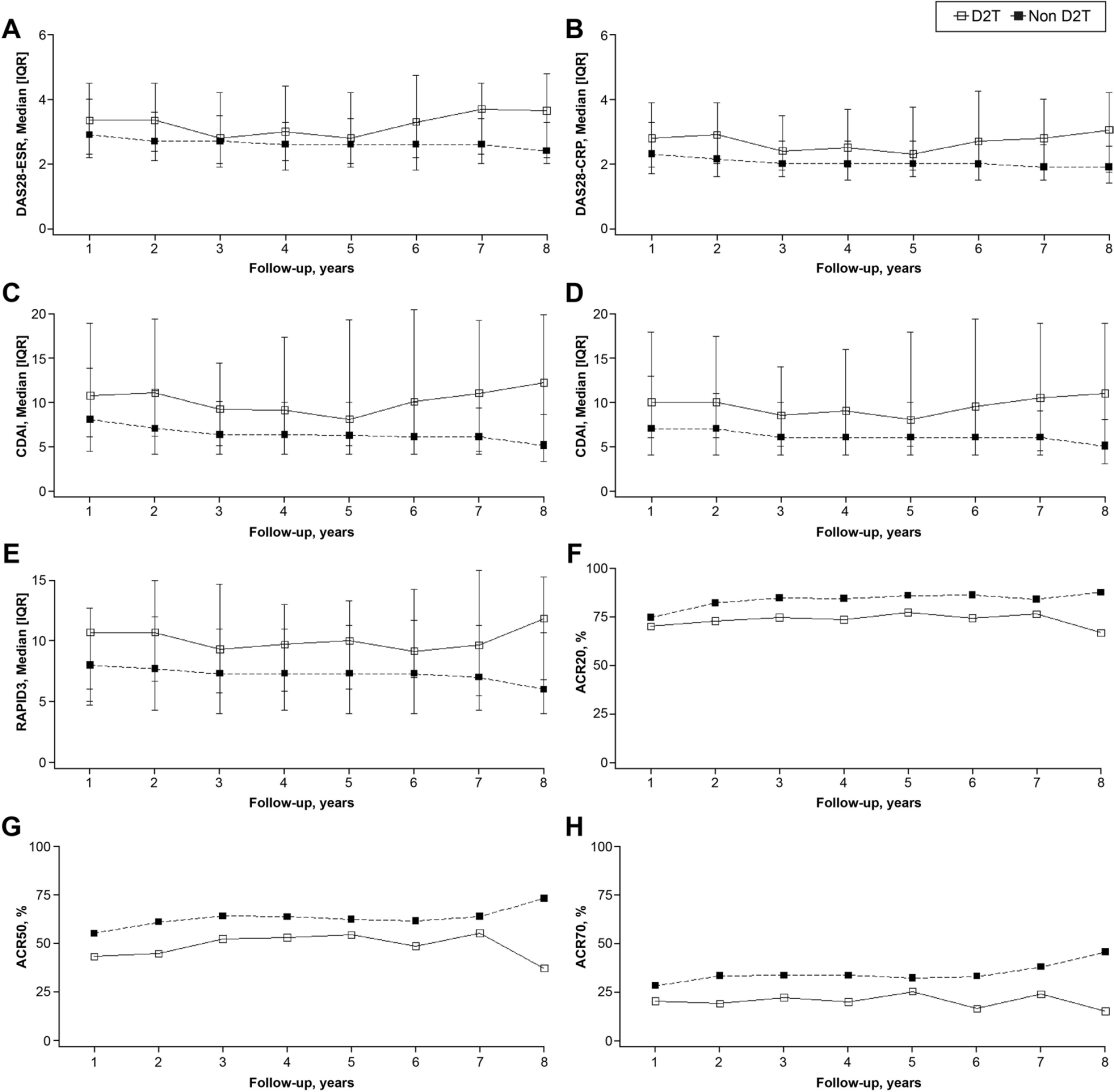
Changes in disease activity between the D2T RA and non-D2T RA groups. **A** DAS28-ESR, **B** DAS28-CRP, **C** SDAI, **D** CDAI, **E** RAPID3, **F** ACR20, **G** ACR50, and **H** ACR70

DAS28-CRP and -ESR, SDAI and CDAI levels were higher in patients with D2T RA compared to patients with non-D2T RA after 8 years (Fig. 3). Furthermore, RAPID3 levels were higher in patients with D2T RA than in those with non-D2T RA after eight years. When comparing the proportions of patients achieving ACR response between the two groups, it was observed that fewer patients with D2T RA achieved ACR 20/50/70 response compared to those with non-D2T RA. Supplementary Table 1 provides a detailed overview of all the values.

### Comparison of adverse events causing drug withdrawal between D2T and non-D2T RA patients

The proportion of adverse events leading to drug withdrawal was 36.1% in the D2T RA group and 32.8% in the non-D2T RA group (*p* = 0.469) (Supplementary Table 2). The 5-year mortality rates were 3.7% for patients with D2T RA and 2.1% for patients with non-D2T RA (*p* = 0.099). The causes of mortality were unknown for 7 patients with D2T RA and 36 patients with non-D2T RA (data not shown). Univariable logistic analysis revealed that age (OR = 1.02, *p* = 0.003), comorbidities (OR = 1.45, *p* = 0.004), and concomitant use of methotrexate (OR = 0.72, *p* = 0.037) were associated with adverse events related to b/tsDMARDs in patients with D2T RA (Supplementary Table 3). However, in the multivariable logistic analysis, no factors were found to be associated with adverse events.

## Discussion

Patients with D2T RA exhibited distinct characteristics compared to those with non-D2T RA, including younger age, longer disease duration, lower patient global assessment scores, higher SDAI and RAPID3 scores, negative RF, and less prior use of csDMARDs such as methotrexate, sulfasalazine, and leflunomide. However, despite these differences, the overall rate of drug retention did not significantly differ between patients with D2T RA and non-D2T RA after an 8-year period. The proportion of adverse events causing drug withdrawal did not differ between the groups, the proportion of drug withdrawals due to inefficacy was higher in patients with D2T RA compared to those with non-D2T RA.

Regarding the risk factors for D2T RA, it was observed that female RA patients and younger patients were more susceptible to D2T, which is consistent with previous data indicating that female sex and earlier onset of RA contribute to higher disease activity or D2T in RA [11, 12]. However, neither BMI nor smoking history showed an association with D2T, in contrast to other studies on D2T RA. Previous research has shown that obesity is associated with a lower rate of achieving remission or low disease activity in RA [13–16]. At this point of discrepancy, the BMIs of the study population differed between the previous studies and the KOBIO data. The mean BMI in the KOBIO data was 22.8 ± 3.5 kg/m^2^, while the proportions of overweight (defined as BMI 25–30 kg/m^2^) or obese (defined as BMI > 30 kg/m^2^) patients were more than 32% and 10%, respectively [17]. Overweight and obesity may contribute to the development of D2T RA, but not in a population with normal BMI values. In addition, the proportion of smoking patients in the KOBIO data was lower than that reported in other cohorts, where it constituted more than 20% of the population, but less than 10% in the Korean RA population including KOBIO data [11, 18, 19]. It is important to consider that the factors predicting D2T RA may differ depending on the characteristics of the RA population.

Patients with D2T RA are known to have a higher frequency of comorbidities, and CVD is reported to be more prevalent among them [20, 21]. The comorbidities associated with RA are not only consequences of systemic inflammation or RA itself but also factors that can lead to non-adherence to treatment or limitations in the choice of medications [22]. The presence of CVD poses challenges in the management of RA, as it restricts the use of certain medications such as GCs, NSAIDs, and several tsDMARDs due to their potential to accelerate atherosclerosis or provoke thrombosis Specifically, tsDMARDs are not recommended for use in patients with CVD risk or in the elderly, as they carry a higher risk of thromboembolism in these high-risk individuals [23, 24]. The presence of combined CVD creates barriers in selecting suitable drugs for managing disease activity in individuals, thereby suggesting a higher likelihood of progression to D2T RA. It is crucial to consider the impact of comorbidities, particularly CVD, on the treatment options and outcomes in patients with RA. The presence of comorbidities can complicate the management of RA and necessitate careful consideration of the risks and benefits of various treatment approaches. Future studies should further explore the relationship between comorbidities, specifically CVD, and the development of D2T RA to enhance our understanding of these complex interactions and inform the development of tailored treatment strategies for this patient population.

A lower proportion of patients with D2T RA had a history of prior use of methotrexate, sulfasalazine, and leflunomide compared to those with non-D2T RA. Furthermore, prior use of these csDMARDs was independently associated with a reduced risk of developing D2T RA, suggesting that incomplete use of csDMARDs may be associated with a higher risk for D2T RA. In other cohorts, the D2T RA group exhibited a higher proportion of patients with contraindications or intolerance to methotrexate and delayed initiation of methotrexate treatment for more than 12 months compared to the non-D2T RA group [11, 25]. Standard recommendations for RA emphasize the early initiation of methotrexate followed by sequential use of other csDMARDs [26, 27]. According to the regulations of the Korean National Health Insurance Services (NHIS), csDMARDs, including methotrexate, should be maintained for at least six months with a minimum of three months of use before initiating b/tsDMARDs. Consequently, a majority of patients in the KOBIO data (92.42% of those with a history of two or more csDMARD treatments) were found to continue using other DMARDs alongside methotrexate. However, there are cases where methotrexate usage may be incomplete due to adverse effects or contraindications, such as combined interstitial lung disease or liver disorders. The incomplete use of methotrexate and other csDMARDs could predict challenges in achieving remission or low disease activity despite sequential use of b/tsDMARDs. These findings underscore the importance of adhering to recommended treatment guidelines, particularly the early and consistent use of methotrexate and other csDMARDs in RA management. Incomplete utilization of these medications may impede the achievement of optimal treatment outcomes, including remission or low disease activity. It is crucial for healthcare providers to address any barriers to csDMARD usage and promote the appropriate and timely initiation of these medications in order to improve treatment responses and prevent the progression to D2T RA.

In contrast to previous studies, the current data did not show an association between GC use and D2T RA. *Giollo *et al. reported that GC use for more than 6 months was associated with D2T RA, while *Yoshii *et al. suggested that treatment with methotrexate at a dose of ≥ 8.7 mg/week without concomitant GCs might aid in withdrawing from D2T RA [12, 25]. It is important to note that the control groups in previous studies consisted of RA patients who were treated with csDMARDs only, whereas the non-D2T RA group in the current study received treatment with b/tsDMARDs. All patients in the current study had relatively higher disease activity, which necessitated the use of low-dose GCs, resulting in more than 85% of patients receiving GC therapy. Among patients with RA who have moderate-to-high disease activity and are being treated with b/tsDMARDs, the role of GCs may be limited. While GC use can lead to various complications of varying severity, it has been demonstrated to be effective in relieving joint pain and preventing joint destruction. Furthermore, tapering GCs can pose a risk of disease flare, and the benefits of low-dose GCs, especially in combination with b/tsDMARD therapy, outweigh any negligible harm associated with their use [28–31]. Taken together, the current findings suggest that the use of csDMARDs may impede the progression to D2T RA, whereas the use of GCs does not appear to play a significant role. Comprehensive understanding and patient care implications warrant further research and clinical observation.

The patterns of drug survival differed between patients with D2T RA and non-D2T RA for each b/tsDMARD. In patients with D2T RA, JAK inhibitors had the highest drug survival rate, while rituximab had the lowest. Conversely, in patients with non-D2T RA, rituximab had the highest drug survival rate, while TNF inhibitors had the lowest. Although the population treated rituximab was very small, patients with D2T RA might have a difficulty to tolerate the several months required for rituximab to take effect, as it has a slow onset of action that can extend beyond 4 months. In addition, rituximab targets and depletes B cells and plasma cells involved in the production and action of autoantibodies in RA, so its efficacy might be limited in patients with D2T RA who have uncontrolled disease activity which was derived from factors other than RF production. Therefore, the observed differences in drug survival rates between D2T RA and non-D2T RA patients suggested specific treatment strategies might be necessary for each of the two disparate groups.

In the present study, disease activity markers remained elevated even after 8 years of treatment with b/tsDMARDs in patients with D2T RA. Despite the use of one b/tsDMARD in combination with a csDMARD, a considerable number of patients with D2T RA were unable to adequately control their symptoms. The current recommendations for the management of RA, which are based on extensive knowledge about the disease, have not yielded satisfactory outcomes in the D2T RA population. Furthermore, the reason why medication cannot be changed despite inadequate symptom control is the limited availability of alternative b/tsDMARDs. Due to the limited options of b/tsDMARDs, patients with D2T RA may be unable to switch to a different medication even if their current treatment is not effectively managing their symptoms. To improve the management of D2T RA, it is crucial to adopt a more comprehensive approach that takes into account the unique challenges and characteristics of this subgroup of patients. By focusing on tailored and targeted interventions, including exploring novel treatment options, it may be possible to identify more effective strategies for symptom relief, improved quality of life, and prevention of complications associated with D2T RA. This calls for further research and collaboration to optimize the management of D2T RA and address the unmet needs of these patients [5, 32].

This study has some limitations. Since the data were extracted retrospectively, it was not possible to control all cases included in the analysis, such as the dosages of methotrexate. Considering the limited number of cases and the characteristics of each medication, we did not separately analyse subcutaneous and intravenous administration methods for drugs offering both options, such as infliximab biosimilar, tocilizumab, abatacept, and golimumab. We were unable to comprehensively categorize the presence or absence of OA and fibromyalgia among the RA patients registered in KOBIO. It is possible that there are RA patients with concomitant OA and fibromyalgia. However, in KOBIO, registration targeted RA patients initiating or switching b/tsDMARDs treatment, leading to the enrollment of RA patients with moderate disease activity or higher. The definition of D2T RA, as per the EULAR criteria, requires patients to be treated according to the EULAR recommendations for RA [2]. While most patients strictly adhere to these recommendations due to reimbursement conditions set by the Korean NHIS, ensuring a minimum of 6 months of csDMARD use before initiating b/tsDMARD therapy, individual treatment histories were not fully controlled in this retrospective study. However, the insurance regulations did provide some boundaries in terms of available treatment options, leading to a somewhat more controlled study population.

## Conclusions

This study identified several important factors associated with D2T RA. RF positivity and prior use of csDMARDs, particularly methotrexate, were found to be associated with a lower risk of developing D2T RA. However, despite similar overall drug retention rates, patients with D2T RA experienced higher rates of medication switching and poorer disease control over an 8-year period. Disease activity markers remained elevated in patients with D2T RA compared to those without. These findings emphasize the challenges faced in managing D2T RA and highlight the need for personalized treatment approaches that consider individual factors such as RF status and previous csDMARD use. Further research is necessary to improve outcomes and develop targeted strategies for symptom relief, improved quality of life, and prevention of complications in patients with D2T RA.

### Supplementary Information

**Additional file 1:** **Supplement Table 1.** Comparison of disease activity markers between D2T and non-D2T group. **Supplementary Table 2.** Comparison of adverse events causing drug withdrawal in D2T and non-D2T group. **Supplementary Table 3.** Logistic regression analysis for adverse events in patients with D2T RA.

## Data Availability

All available data are reported in the manuscript and supplementary file.

## Abbreviations

ACR: American College of Rheumatology
anti-CCP: Anti-cyclic citrullinated peptide
bDMARDs: Biologic disease-modifying anti-rheumatic drugs
BMI: Body mass index
CDAI: Clinical Disease Activity Index
CRP: C-reactive protein; csDMARDs, conventional synthetic DMARDs
CVD: Cardiovascular disease
DAS28: Disease activity score 28 joints
D2T: Difficult-to-treat
ESR: Erythrocyte sedimentation rate
EULAR: European League Against Rheumatism
GCs: Glucocorticoids
IQR: Interquartile range
IRB: Institutional review boards
JAK: Janus kinase
KOBIO registry: Korean College of Rheumatology Biologics registry
NHIS: National Health Insurance Services
RAPID3: Routine assessment of patient index data 3
RF: Rheumatoid factor
RA: Rheumatoid arthritis
SD: Standard deviation
SDAI: Simplified Disease Activity Index
tsDMARDs: Targeted synthetic disease-modifying anti-rheumatic drugs
TNF: Tumor necrosis factor
