# Supplementary material for: Unveiling difficult-to-treat rheumatoid arthritis: long-term impact of biologic or targeted synthetic DMARDs from the KOBIO registry
Source: Arthritis Res Ther. 2023 Sep 19;25:174. doi: 10.1186/s13075-023-03165-w (PMC10507947; doi:10.1186/s13075-023-03165-w)
Supplement: Supplementary file 1 — Additional file 1: Supplement Table 1. Comparison of disease activity markers between D2T and non-D2T group. Supplementary Table 2. Comparison of adverse events causing drug withdrawal in D2T and non-D2T group. Supplementary Table 3. Logistic regression analysis for adverse events in patients with D2T RA. [file 13075_2023_3165_MOESM1_ESM.docx]

Supplement Table 1. Comparison of disease activity markers between D2T and non-D2T groups

|  |  | D2T | Non-D2T | *p-*value |
| --- | --- | --- | --- | --- |
| 1st | N | 238 | 1,922 |  |
|  | ESR, mm/hr | 20 [8, 43] | 19.5 [7.5, 38] | <0.001 |
|  | CRP, mg/dL | 0.21 [0.03, 0.99] | 0.14 [0.03, 0.73] | <0.001 |
|  | DAS28-ESR | 3.35 [2.30, 4.50] | 2.90 [2.20, 4.00] | <0.001 |
|  | DAS28-CRP | 2.80 [1.90, 3.90] | 2.30 [1.70, 3.30] | <0.001 |
|  | SDAI | 10.8 [6.0, 19.0] | 8.0 [4.4, 13.9] | <0.001 |
|  | CDAI | 10.0 [6.0, 18.0] | 7.0 [4.0, 13.0] | <0.001 |
|  | RAPID3 | 10.7 [6.0, 5.0] | 8.0 [4.7, 12.7] | <0.001 |
|  | ACR20 response | 161 (70.0) | 1,398 (74.6) | 0.137 |
|  | ACR50 response | 99 (43.0) | 1,033 (55.1) | 0.001 |
|  | ACR70 response | 46 (20.0) | 526 (28.1) | 0.010 |
| 2nd | N | 206 | 1,610 |  |
|  | ESR, mm/hr | 20 [8, 38.5] | 18 [7, 35] | <0.001 |
|  | CRP, mg/dL | 0.20 [0.03, 1.03] | 0.13 [0.03, 0.53] | <0.001 |
|  | DAS28-ESR | 3.35 [2.40, 4.50] | 2.70 [2.10, 3.60] | <0.001 |
|  | DAS28-CRP | 2.90 [2.00, 3.90] | 2.15 [1.60, 2.90] | <0.001 |
|  | SDAI | 11.1 [6.1, 19.5] | 7.0 [4.1, 11.3] | <0.001 |
|  | CDAI | 10.0 [6.0, 17.5] | 7.0 [4.0, 11.0] | <0.001 |
|  | RAPID3 | 10.7 [6.70, 15.0] | 7.7 [4.3, 12.0] | <0.001 |
|  | ACR20 response | 147 (72.8) | 1,291(82.1) | 0.001 |
|  | ACR50 response | 90 (44.6) | 958 (60.9) | <0.001 |
|  | ACR70 response | 38 (18.8) | 521 (33.1) | <0.001 |
| 3rd | N | 170 | 1,309 |  |
|  | ESR, mm/hr | 19 [6, 39] | 17 [6, 32] | 0.219 |
|  | CRP, mg/dL | 0.2 [0.03, 1.00] | 0.11 [0.03, 0.5] | 0.008 |
|  | DAS28-ESR | 2.80 [2.00, 4.20] | 2.70 [1.90, 3.50] | 0.001 |
|  | DAS28-CRP | 2.40 [1.80, 3.50] | 2.00 [1.60, 2.70] | <0.001 |
|  | SDAI | 9.2 [5.1, 14.5] | 6.3 [4.1, 10.1] | <0.001 |
|  | CDAI | 8.5 [5.0, 14.0] | 6.0 [4.0, 10.0] | <0.001 |
|  | RAPID3 | 9.3 [5.7, 14.7] | 7.3 [4.0, 11.0] | <0.001 |
|  | ACR20 response | 123 (74.6) | 1,092 (84.8) | 0.001 |
|  | ACR50 response | 86 (52.1) | 825 (64.1) | 0.003 |
|  | ACR70 response | 36 (21.8) | 430 (33.4) | 0.003 |
| 4th | N | 135 | 997 |  |
|  | ESR, mm/hr | 19 [6, 50] | 16 [5, 29] | 0.017 |
|  | CRP, mg/dL | 0.26 [0.03, 1.00] | 0.10 [0.03, 0.42] | <0.001 |
|  | DAS28-ESR | 3.00 [2.10, 4.40] | 2.60 [1.80, 3.30] | <0.001 |
|  | DAS28-CRP | 2.50 [1.90, 3.70] | 2.00 [1.50, 2.70] | <0.001 |
|  | SDAI | 9.1 [6.0, 17.4] | 6.3 [4.1, 10.0] | <0.001 |
|  | CDAI | 9.0 [6.0, 16.0] | 6.0 [4.0, 9.0] | <0.001 |
|  | RAPID3 | 9.7 [5.9, 13.0] | 7.3 [4.3, 11.0] | <0.001 |
|  | ACR20 response | 97 (73.5) | 825 (84.3) | 0.002 |
|  | ACR50 response | 70 (53.0) | 623 (63.6) | 0.018 |
|  | ACR70 response | 26 (19.7) | 327 (33.4) | 0.002 |
| 5th | N | 94 | 704 |  |
|  | ESR, mm/hr | 13 [5, 43] | 16 [6, 31] | 0.929 |
|  | CRP, mg/dL | 0.17 [0.03, 0.78] | 0.1 [0.03, 0.49] | 0.119 |
|  | DAS28-ESR | 2.80 [1.90, 4.20] | 2.60 [2.00, 3.40] | 0.055 |
|  | DAS28-CRP | 2.30 [1.80, 3.75] | 2.00 [1.60, 2.70] | 0.001 |
|  | SDAI | 8.1 [5.1, 19.4] | 6.2 [4.1, 10.0] | 0.001 |
|  | CDAI | 8.0 [5.0, 18.0] | 6.0 [4.0, 10.0] | <0.001 |
|  | RAPID3 | 10.0 [6.0, 13.3] | 7.3 [4.0, 11.3] | 0.001 |
|  | ACR20 response | 71 (77.2) | 582 (86) | 0.027 |
|  | ACR50 response | 50 (54.4) | 422 (62.3) | 0.14 |
|  | ACR70 response | 23 (25.0) | 217 (32.1) | 0.171 |
| 6th | N | 62 | 442 |  |
|  | ESR, mm/hr | 17.5 [4, 48] | 16 [6, 32] | 0.451 |
|  | CRP, mg/dL | 0.17 [0.02, 0.98] | 0.1 [0.03, 0.38] | 0.277 |
|  | DAS28-ESR | 3.30 [2.20, 4.75] | 2.60 [1.80, 3.30] | <0.001 |
|  | DAS28-CRP | 2.70 [1.90, 4.25] | 2.00 [1.50, 2.60] | <0.001 |
|  | SDAI | 10.1 [6.0, 20.6] | 6.1 [4.0, 10.0] | <0.001 |
|  | CDAI | 9.5 [6.0, 19.5] | 6.0 [4.0, 9.5] | <0.001 |
|  | RAPID3 | 9.2 [7.0, 14.3] | 7.3 [4.0, 11.7] | 0.002 |
|  | ACR20 response | 46 (74.2) | 367 (86.2) | 0.015 |
|  | ACR50 response | 30 (48.4) | 262 (61.5) | 0.049 |
|  | ACR70 response | 10 (16.1) | 140 (32.9) | 0.008 |
| 7th | N | 39 | 271 |  |
|  | ESR, mm/hr | 21 [8, 56] | 17 [8, 31] | 0.299 |
|  | CRP, mg/dL | 0.30 [0.04, 1.15] | 0.11 [0.03, 0.40] | 0.044 |
|  | DAS28-ESR | 3.70 [2.30, 4.50] | 2.60 [2.00, 3.40] | 0.004 |
|  | DAS28-CRP | 2.80 [1.80, 4.00] | 1.90 [1.50, 2.60] | 0.001 |
|  | SDAI | 11.0 [4.4, 19.3] | 6.1 [4.0, 9.4] | <0.001 |
|  | CDAI | 10.5 [4.5, 19.0] | 6.0 [4.0, 9.0] | <0.001 |
|  | RAPID3 | 9.7 [5.5, 15.9] | 7.0 [4.3, 11.3] | 0.022 |
|  | ACR20 response | 29 (76.3) | 220 (84.0) | 0.241 |
|  | ACR50 response | 21 (55.3) | 167 (63.7) | 0.313 |
|  | ACR70 response | 9 (23.7) | 99 (37.8) | 0.091 |
| 8th | N | 28 | 147 |  |
|  | ESR, mm/hr | 19 [7, 56] | 18 [7, 31] | 0.476 |
|  | CRP, mg/dL | 0.15 [0.04, 1.00] | 0.08 [0.03, 0.39] | 0.132 |
|  | DAS28-ESR | 3.70 [2.20, 4.80] | 2.40 [2.00, 3.30] | 0.008 |
|  | DAS28-CRP | 3.10 [1.80, 4.20] | 1.90 [1.40, 2.60] | 0.002 |
|  | SDAI | 12.2 [5.6, 20.0] | 5.1 [3.3, 8.6] | <0.001 |
|  | CDAI | 11.0 [5.5, 19.0] | 5.0 [3.0, 8.0] | <0.001 |
|  | RAPID3 | 11.9 [6.8, 15.3] | 6.0 [4.0, 10.7] | 0.002 |
|  | ACR20 response | 18 (66.7) | 127 (87.6) | 0.006 |
|  | ACR50 response | 10 (37.0) | 106 (73.1) | <0.001 |
|  | ACR70 response | 4 (14.8) | 66 (45.5) | 0.003 |

Data were presented with median [interquatile range]or N(%).

**P*-value was calculated from the Wilcoxon Rank-Sum test or Chi-squared test.

Supplementary Table 2. Comparison of adverse events causing drug withdrawal in D2T and non-D2T groups

| Variable | Total | D2T | | Non-D2T | *p-value* |
| --- | --- | --- | --- | --- | --- |
| Adverse events, N (%) | 340 (33.2) | 44 (36.1) | | 296 (32.8) | 0.469 |
| Acute coronary syndrome | 2 |  | | 2 |  |
| Atypical mycobacterial infection pulmonary | 7 | 2 | | 5 |  |
| Cerebrovascular accident | 2 |  | | 2 |  |
| Conception | 9 | 1 | | 8 |  |
| Congestive heart failure | 1 |  | | 1 |  |
| Death | 21 | 3 | | 18 |  |
| Fungal infection | 1 |  | | 1 |  |
| Hepatitis B reactivation | 5 |  | | 5 |  |
| Herpes zoster infection | 9 |  | | 9 |  |
| Infusion/injection reaction | 106 | 11 | | 95 |  |
| Interstitial lung disease | 7 |  | | 7 |  |
| Lymphoma | 4 |  | | 4 |  |
| Malignancy Other | 3 | 1 | | 2 |  |
| Malignancy Solid | 20 | 6 | | 14 |  |
| Mycobacteria tuberculosis infection extra-pulmonary | 1 |  | | 1 |  |
| Mycobacteria tuberculosis infection pulmonary | 8 | 1 | | 7 |  |
| Neutropenia | 3 |  | | 3 |  |
| Other infection | 54 | 5 | | 49 |  |
| Others | 113 | 17 | | 96 |  |
| Pneumocystis jiroveci infection | 1 |  | | 1 |  |
| Psoriasis | 4 |  | | 4 |  |
| Pulmonary embolism | 1 |  | | 1 |  |
| SLE | 1 |  | | 1 |  |
| Transaminitis only | 7 | 2 | | 5 |  |
| Total Adverse events cases | 390 | | 49 | 341 |  |

KOBIO: Korean College of Rheumatology Biologics & Targeted therapy, D2T: difficult to treat.

*P*-values are calculated using chi-square test.

Supplementary Table 3. Logistic regression analysis for adverse events in patients with D2T RA

| **Variable** | **Univariable** | | **Multivariable** | |
| --- | --- | --- | --- | --- |
|  | **OR (95% CI)** | ***p*** | **Adjusted OR (95% CI)** | ***p*** |
| D2T | 1.24 (0.84, 1.83) | 0.280 | 1.17 (0.78, 1.77) | 0.455 |
| Age | 1.02 (1.01, 1.03) | **0.003** | 1.01 (1.00, 1.02) | 0.066 |
| Sex, female | 1.01 (0.73, 1.40) | 0.955 | 1.25 (0.78, 2.01) | 0.348 |
| BMI | 0.98 (0.95, 1.02) | 0.337 | 0.97 (0.93, 1.01) | 0.149 |
| Current, ex- smoker | 1.17 (0.85, 1.61) | 0.333 | 1.38 (0.87, 2.17) | 0.171 |
| Disease duration | 1.01 (1.00, 1.03) | 0.185 | 1.01 (0.99, 1.02) | 0.607 |
| Patient global assessment | 1.01 (0.95, 1.08) | 0.723 | 0.95 (0.86, 1.05) | 0.330 |
| Physician global assessment | 1.06 (0.99, 1.14) | 0.094 | 1.10 (1.00, 1.21) | 0.060 |
| DAS28-ESR | 1.06 (0.95, 1.19) | 0.297 | 1.06 (0.80, 1.41) | 0.690 |
| DAS28-CRP | 1.03 (0.92, 1.16) | 0.573 | 1.07 (0.70, 1.63) | 0.764 |
| SDAI | 1.00 (0.99, 1.01) | 0.566 | 0.96 (0.90, 1.02) | 0.210 |
| CDAI | 1.01 (0.99, 1.02) | 0.362 | 1.04 (0.98, 1.09) | 0.200 |
| RAPID3 | 1.00 (0.98, 1.03) | 0.827 | 0.99 (0.96, 1.02) | 0.534 |
| Comorbidities* | 1.45 (1.13, 1.86) | **0.004** | 1.31 (0.99, 1.75) | 0.061 |
| RF positivity | 1.21 (0.85, 1.71) | 0.283 | 1.18 (0.80, 1.73) | 0.399 |
| Anti-CCP Ab positivity | 1.00 (0.70, 1.43) | 0.992 | 0.92 (0.62, 1.36) | 0.670 |
| Prior use of methotrexate | 0.65 (0.41, 1.04) | 0.071 | 0.85 (0.48, 1.5) | 0.564 |
| Prior use of sulfasalazine | 1.04 (0.80, 1.34) | 0.791 | 1.08 (0.83, 1.41) | 0.564 |
| Prior use of leflunomide | 0.96 (0.74, 1.23) | 0.723 | 0.90 (0.70, 1.17) | 0.443 |
| Concomitant methotrexate | 0.72 (0.53, 0.98) | **0.037** | 0.83 (0.57, 1.21) | 0.328 |
| Concomitant corticosteroid | 0.87 (0.62, 1.22) | 0.418 | 0.86 (0.60, 1.22) | 0.388 |

bDMARDs: biologic disease modifying anti-rheumatic drugs, tsDMARDs: target synthetic disease modifying anti-rheumatic drugs, D2T: difficult to treat, OR: odds ratio, CI: confidence interval, BMI: body mass index, DAS: disease activity score, ESR: erythrocyte sedimentation rate, CRP: C-reactive protein, SDAI: simplified disease activity index, CDAI: clinical disease activity index, RAPID3: routine assessment of patient index data 3, RF: rheumatoid factor, Anti-CCP Ab: anti-citrullinated protein antibody, csDMARDs: conventional-synthetic disease modifying anti-rheumatic drugs.

*Comorbidities include hypertension, diabetes mellitus, cardiovascular diseases, and cancer.
Values are calculated using logistic regression model. Bold values indicate significant *p*-values.
